# Nobel prize in physiology or medicine 2021, receptors for temperature and touch: Implications for hematology

**DOI:** 10.1002/ajh.26407

**Published:** 2021-11-20

**Authors:** Immacolata Andolfo, Seth L. Alper, Achille Iolascon

**Affiliations:** ^1^ Department of Molecular Medicine and Medical Biotechnologies University Federico II of Naples Naples; ^2^ CEINGE, Biotecnologie Avanzate Naples; ^3^ Renal Division and Molecular and Vascular Medicine Division, Beth Israel Deaconess Medical Center and Department of Medicine Harvard Medical School Boston Massachusetts USA

The Nobel Prize in Physiology or Medicine 2021 was awarded jointly to David Julius and Ardem Patapoutian for their discoveries of receptors for temperature and touch.

David Julius, Professor of Physiology at UCSF, studied mechanisms by which we sense heat, cold, and chemical irritants. Julius and colleagues developed a novel genome‐wide screen for elevations in intracellular [Ca^2+^] in response to chemicals we perceive as “hot.” As reported in 1997, the screen identified cDNAs encoding TRPV1, a heat sensor acting as receptor for vanilloid compounds such as capsaicin.^1^ This discovery was accompanied by the realization that TRPV1 is part of a large TRP gene superfamily related to the Transient Receptor Potential (*trp*) gene of the *Drosophila* visual transduction pathway. The discovery of TRPV1 was crucial to understanding how temperature variation induces electrical signals in the peripheral nervous system and helps process those signals centrally.

David Julius shared the Nobel award with Ardem Patapoutian, Professor of Neuroscience at Scripps Research Institute, La Jolla, California, USA, whose research efforts focused on cellular sensors that detect mechanical stimuli and transduce them as intracellular and juxtacrine chemical signals. In 2010, Patapoutian and colleagues reported their discovery of the ion channels PIEZO1 and PIEZO2 (after *píesi*, Greek for “pressure”), using a novel cellular screen for mechanosensitive elevations in intracellular [Ca^2+^].^2^ PIEZO1 and PIEZO2 are plasma membrane ion channels susceptible to activation by lateral stretch of the lipid bilayer membrane. This lateral stretch may arise from cell poking or indentation, cell matrix stretching, hydrostatic pressure change, osmotic pressure change (manifest as cell volume change), and also isovolumic cell shape change.^2^ Additional perturbations that activate PIEZO channels include those involved in our tactile sensation of light touch, pressure, and pain, as well as cellular responses to fluid flow and to (ultra)sound. Further studies have demonstrated that PIEZO1 and PIEZO2 regulate other crucial physiological processes including blood pressure, respiration, and urinary bladder control. Endogenous and exogenous chemical ligands can directly activate PIEZO channels or modulate their sensitivity to mechanical stimuli.

The 2021 Nobel Prize in Physiology or Medicine is relevant to hematology in several ways. The first connection is between TRPV1 and sickle cell disease (SCD). Pain is the principal cause of emergency department visits, hospitalizations, and everyday suffering in SCD. Both patients and transgenic mice with SCD display chronic mechanical, thermal, and chemical hypersensitivity, which may be mediated by TRPV1 channel signaling.^3^, ^4^ Indeed, the selective TRPV1 antagonist, A‐425619, has been reported to reverse mechanical sensitization and to attenuate mechanical behavioral hypersensitivity in SCD mice.^4^ Oral administration to SCD mice of the TRPV1 agonist, capsaicin, which activates somatosensory nerves through TRPV1 binding, was shown to dramatically alleviate acute vaso‐occlusive events and to significantly reduce ensuing chronic liver and kidney damage.^4^ Thus, pharmacological manipulation of TRPV1 activity may provide a promising approach to treat both the pain symptoms and the ischemic damage of SCD.

The connections between the mechanoreceptor PIEZO1 and hemolytic anemias are multiple. In 2012–2013, PIEZO1 was identified by exome sequencing as a causative gene of both isolated and syndromic forms of dehydrated hereditary stomatocytosis (DHS, also known as hereditary xerocytosis).^5^, ^6^ DHS is an autosomal dominant hemolytic anemia characterized by high reticulocyte count, tendency to macrocytosis, and mild jaundice. It is characterized by many other variably penetrant clinical features, including perinatal edema, severe thromboembolic complications after splenectomy, and hepatic iron overload (Figure 1). The phenotype of DHS patients ranges from asymptomatic to mild anemia.^7^ The main characteristic of the erythrocyte is cell dehydration caused by the loss of cellular K, which can be assessed by atomic absorption spectroscopy, osmotic fragility, or ektacytometry. PIEZO1 mutations in DHS lead to a gain‐of‐function (GoF) phenotype, either by slowing inactivation kinetics of the ion channel and/or by facilitating channel opening in response to physiological stimuli (Figure 1).^7^, ^8^ PIEZO1 is believed to be the key sensor of red cell membrane curvature, signaling modulation of intracellular ion content, and cell volume as a function of mechanical forces and constraints in capillaries and venules.

**FIGURE 1:**
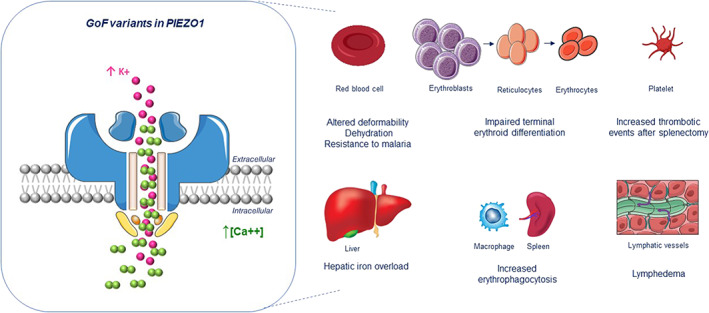
Schematic representation of PIEZO1 and its implications in dehydrated hereditary stomatocytosis. *Left*: Schematic representation of homotrimeric PIEZO1 in the plasma membrane. Gain‐of‐function (GoF) variants in PIEZO1 can cause dehydrated hereditary stomatocytosis (DHS). The GoF variants of DHS delay inactivation of the activated mechanoreceptor, leading to increased net K^+^ efflux and increased Ca^2+^ influx. *Right*: Tissues and cells implicated in the pathophysiology of PIEZO1‐related DHS: red blood cells can be dehydrated and less deformable; late stages of erythroid differentiation are impaired, including reticulocyte formation; platelets are implicated by an unknown mechanism in the increased thrombotic risk post‐splenectomy; liver is affected by severe iron overload related to hepcidin suppression; macrophage overactivation can increase erythropoiesis despite late erythropoietic delay; lymphatic dysfunction is implicated in prenatal and perinatal edema [Color figure can be viewed at wileyonlinelibrary.com]

Red cell dehydration is an important factor in SCD pathophysiology. Three major ion transport pathways are involved in sickle cell dehydration: the K‐Cl cotransporters (KCCs), the Gardos channel (KCNN4), and Psickle, the deoxyhemoglobin S polymerization‐induced cation permeability found only in red cells of SCD patients, and most likely mediated by PIEZO1.^9^ Additionally, PIEZO1 may mediate a major fraction of the increased cation permeability of hereditary spherocytosis RBCs.^10^

The PIEZO1 E756del variant, present in up to 30% of individuals of African ancestry, was first characterized by delayed PIEZO1 ion channel inactivation and by very mild dehydration of RBC,^11^ and was associated with RBC dehydration in patients with SCD.^12^ However, subsequent studies have failed to document increased red cell dehydration^13^, ^14^ or increased Psickle activity^14^ in SCD red cells heterozygous or homozygous for PIEZO1 E756del.

The PIEZO1 E756del variant was also associated with resistance to erythroid invasion by malarial parasites in vitro and in mice,^11^ a finding subsequently confirmed by the association between the PIEZO1 E756del and decreased severity of *Plasmodium falciparum* malaria in human patients, possibly reflecting decreased red cell surface expression of the plasmodial virulence factor, PfEMP.^13^ The protection against severe malaria afforded by the PIEZO1 variant was not additive to that afforded by sickle trait.

PIEZO1 also influences erythropoiesis: PIEZO1 activation during erythroid differentiation slowed differentiation and reticulocyte maturation.^15^

Recent evidence highlights a role for PIEZO1 in regulation of iron metabolism. DHS patients can exhibit hyperferritinemia (and even hemosiderosis) accompanied by very low values of plasma hepcidin. Overexpression and chemical activation in hepatoma cell lines of the R2456H and R2488Q PIEZO1 GoF mutants induced stronger Ca^2+^ influx than in cells expressing WT PIEZO1. The increased Ca^2+^ signal was associated with ERK phosphorylation, inhibition of the BMP/SMADs pathway, and in decreased expression of *HAMP*, encoding hepcidin.^16^ PIEZO1 involvement in iron metabolism was further confirmed in constitutive and in macrophage‐specific transgenic PIEZO1 GoF mice. By 1 year of age, these mice develop severe hepatic hemosiderosis with elevated serum ferritin and transferrin saturation, accompanied by increased erythrophagocytosis, erythropoiesis, and erythroferrone.^17^ Increased serum ferritin and transferrin saturation were also observed in the over‐40 age subgroup of African Americans carrying the E756del variant in PIEZO1.

The discovery of *PIEZO1* as the major cause of DHS has increased our knowledge of PIEZO1 functions in the erythroid system and in systemic iron metabolism. The links between red cell physiology, iron metabolism, and the sense of touch embodied by PIEZO1 were previously unforeseen. The link between TRPV1‐mediated nociception and pain in SCD, though itself not surprising, revealed a relationship quite distinct from that initially hypothesized. These findings illustrate the close, mutual dependence of basic and translational research in our ongoing investigation of the pathobiology and treatment of human disease. Indeed, continued study of temperature receptors and mechanoreceptors may soon identify new druggable targets for the still challenging treatment of anemia and chronic iron overload.

## CONFLICT OF INTEREST

Immacolata Andolfo and Achille Iolascon declare no conflict of interest. Seth L. Alper is a consultant for and receives research support from Quest Diagnostics, Inc.

## Data Availability

Not applicable.
